# Critical appraisal of how COVID-19 infection and imposed lockdowns have impacted gastroesophageal reflux: A review

**DOI:** 10.1097/MD.0000000000038074

**Published:** 2024-05-10

**Authors:** Hafez Al-Momani, Iman Aolymat, Sameer Al Haj Mahmoud

**Affiliations:** a Department of Microbiology, Pathology and Forensic Medicine, Faculty of Medicine, The Hashemite University, Zarqa, Jordan; b Department of Anatomy, Physiology & Biochemistry, Faculty of Medicine, The Hashemite University, Zarqa, Jordan; c Department of Basic Medical Science, Faculty of Medicine, Al-Balqa’ Applied University, Al-Salt, Jordan.

**Keywords:** COVID-19, extraesophageal reflux (EER), gastroesophageal reflux disease (GERD), microaspiration, SARS-CoV-2

## Abstract

Previous literature has demonstrated that COronaVIrus Disease of 2019 (COVID-19) impacts an individual gastrointestinal tract (GIT), causing symptoms like nausea, diarrhea, and loss of appetite. Severe acute respiratory syndrome coronavirus RNA has been discovered in the stool of infected individuals in earlier research. It was discovered that severe acute respiratory syndrome coronavirus was significantly expressed in the GIT, indicating that the virus can also infect the digestive system. Angiotensin-converting enzyme 2 functions as the viral receptor. The chronic illness known as gastroesophageal reflux disease (GERD) is typified by frequent reflux of stomach acid into the esophagus. By triggering the sensitized esophageal-bronchial neuronal circuit or aspirating into the airways (microaspiration), GER exacerbates respiratory diseases. Aspiration is a well-known risk to be considered when treating patients in intensive care units. Strong genetic correlations have been identified between COVID-19 infection and GERD susceptibility, suggesting a shared genetic basis for both conditions. Nonetheless, even though GERD, extraesophageal reflex, and COVID-19 have a number of significant risk factors and exhibit similar symptoms, the relationship between these illnesses has not yet been examined in depth. This review is the first of its kind to critically examine the association between the COVID-19 epidemic and GER and its associated diseases. The key objective of this work is to promote the creation of prevention plans, treatment plans, and guidelines while also enhancing and optimizing our understanding of the relationship between COVID-19 and GERs.

## 1. Introduction

The COVID-19 pandemic has impacted tens of millions of people worldwide. The virus that causes COVID-19 is the recently discovered severe acute respiratory syndrome coronavirus (SARS-CoV-2), which enters host cells through the angiotensin-converting enzyme 2 (ACE2) and transmembrane serine protease 2.^[1,2]^ When the virus infects human hosts, it causes an overreaction of the immune system and symptoms similar to pneumonia. If untreated, this can be fatal.^[2]^ Despite the successful development of a COVID-19 vaccine,^[3]^ little is known about the risk factors that contribute to COVID-19 susceptibility. A number of epidemiological studies conducted in the past have revealed an increase in GIT disease and associated symptoms with COVID-19.^[4–6]^

GI symptoms can emerge in those suffering from COVID-19, as the SARS-CoV-2 disrupts the normal intestinal mucosa. A pooled prevalence of GI symptoms (17.6%) was found in data from a meta-analysis that included 60 investigations with 4243 patients.^[7]^ Anorexia was the most frequently reported symptom (26.8%), followed by diarrhea (12.5%), nausea and vomiting (10.2%), and stomach discomfort (9.2%). GI symptoms were frequently associated with the severity of COVID-19.^[8]^ It interesting to note that there was a 70% increased probability of SARS-CoV-2 severity when GI symptoms were evident upon presentation.^[9]^

Gastroesophageal reflux disease (GERD) is one of the most prevalent GIT disorders. The term “GERD” refers to a medical disorder that arises when stomach acid reflux results in uncomfortable symptoms and/or complications.^[10]^ Heartburn and reflux are the hallmark symptoms of GERD and are sometimes called “typical GERD.”^[10–12]^ Extraesophageal reflux (EER) can manifest in an atypical manner. Chronic cough, asthma, laryngitis, and tooth erosions are common EER signs.^[13,14]^ Meanwhile, direct (aspiration) and indirect (neurally mediated) pathways are 2 possible routes through which EER can exacerbate respiratory symptoms and disorders.^[15]^ The contents of the stomach can thus enter and come into contact with the larynx or airway. In turn, this reflux may directly irritate the throat, or it may trigger a vagal reflex arc that causes coughing and/or bronchospasm.

Even though GERD, EER, and COVID-19 have a number of significant risk factors and exhibit similar symptoms, the relationship between these illnesses has not been thoroughly investigated.^[16]^ For example, smoking and obesity have been shown to be risk factors for COVID-19 and GERD.^[17]^ Moreover, a genetic correlation was identified between the likelihood of hospitalization from COVID-19 and the susceptibility to GERD. The results of the Mendelian randomization trial indicated that there may be a causative relationship between the 2.^[18]^ The number of studies in the literature on the possible effect of COVID-19 infection on GER is limited. Thus, it is thought that this literature review will make an important contribution to the literature. This review is the first of its kind to critically examine the link between the COVID-19 epidemic and GERD. The key objective of this critical review is to promote the creation of prevention plans, treatment plans, and guidelines while also enhancing and optimizing our understanding of the connection between COVID-19 and GERs.

## 2. Gastrointestinal and COVID-19: pathophysiology and pathology

By attaching to its receptor, ACE2, the homotrimer Spike protein helps to connect the viral envelope to host cells by protruding from the surface of the COVID-19 virus.^[19]^ It was first believed that this receptor expression in cells of the lower respiratory tract explained the respiratory symptoms and lung disease associated with the COVID-19 pandemic.^[20]^ Subsequent reports revealed that ACE2 is significantly expressed in the colonocytes of the colon, the lining enterocytes from the ileum, and the esophageal epithelium.^[21]^ The discovery of the virus in the GI lumen near its matching receptor provides compelling evidence that COVID-19 can actively replicate, infect, and cause enteric disease, perhaps resulting in fecal transmission.^[22]^ Moreover, an unexpected finding has also been revealed in that 64% of COVID-19 patients continued to test positive for viral RNA in the feces once the pharyngeal test was showing negative.^[23]^ Tests conducted on RT-qPCR confirmed the presence of fecal SARS-CoV-2 RNA content, even when pharyngeal swabs were negative.^[24]^ Stools may thus serve as a transmission route for the virus.^[25–27]^

From the esophagus to the distal colon, every segment of the gut is affected by the GI topographic disease caused by the SARS-CoV-2.^[28]^ There may be edema, lymphocyte infiltrations, plasma cells, and squamous epithelium infiltrations in the stomach and esophagus.^[29]^ Different degrees of necrosis, degeneration, and epithelial shedding can be seen in the GI mucosa.^[30]^ Multiple previous studies have reported the viral tropism of COVID-19 to the GI mucosa. In the cytoplasm of gastric, duodenal, and colonic glandular epithelial cells (and in the stools), the virus and nucleocapsid proteins were found.^[31,32]^ These pathological results confirm the virus ability to enter the GI tract and cause damage while triggering the host immune system to fight the invader.

## 3. GERD and COVID-19 lockdown measures

Due to the high transmissibility of COVID-19, several governments implemented “lockdown” measures in order to reverse the exponential development trajectories of the virus.^[33]^ A lockdown can be defined as a collection of compulsory and broadly implemented measures that include limitations on the normal course of social and economic activity, which are intended to prevent the spread of COVID-19. Prior research indicated that patients with chronic illnesses such as diabetes, mental health issues, and hypertension experienced changes in their daily lives and health as a result of the lockdown implemented to control COVID-19.^[34,35]^ However, Only 1 study has examined the changes in GERD synonyms during the COVID-19 epidemic and lockdown.^[36]^

This research^[36]^ involved 210 people with preexisting GERD diagnoses and the findings highlighted a statistically significant increase in the frequency of GERD symptoms during the pandemic. Most participants in Al-Momani, Balawi, Almasri, AlGhawrie, Ibrahim, Adli, Balawi and Haj Mahmoud^[36]^ study reported an increased frequency of heartburn and regurgitation during the pandemic, along with increased difficulties falling asleep and increased medicine need for their symptoms. These findings are in line with those revealed by Oliviero, Ruggiero, D’Antonio, Gagliardi, Nunziata, Di Sarno, Abbatiello, Di Feo, De Vivo and Santonicola,^[37]^ and Lee, Huo and Huang,^[38]^ which found that some GI symptoms, such as functional dyspepsia and irritable bowel syndrome, were exacerbated during COVID-19 lockdown.

The increase in GERD symptoms experienced during the pandemic and subsequent lockdowns may be due to lifestyle risks, such as smoking and drinking more coffee, which were found to be more prevalent during the pandemic than before.^[39]^ Small adjustments such as eating bigger lunches at home (as opposed to smaller lunches in the office) can also impact reflux because many people are changing their behavior due to the COVID-19 epidemic and lockdown measures. People were also eating and lying down more frequently.^[40]^ A systematic review of longitudinal studies found that, throughout lockdown, there was a significant change in eating habits that was characterized by more frequent snacking and a preference for ultra-processed and sweet foods over fruits, vegetables, and fresh food.^[41]^ Consuming fat makes reflux symptoms seem worse.^[42]^ Furthermore, an increase in alcohol consumption was revealed in many countries.^[43]^ GER is typically brought on by alcohol, wine and beer in the hour following consumption.^[42,44]^ Furthermore, prior studies have revealed that there is a pressure reduction in the muscle ring between the esophagus and stomach (sometimes referred to as the lower esophageal sphincter [LES]) and an increase in esophageal acid exposure when food is eaten that is high in fats, such as chocolate and sweets.^[44–46]^ There was thus a reduction in adherence to healthy eating regimes. These findings are important in addressing the risk of developing reflux, which may be a predisposing factor for GER.

Increased body weight has also been linked to higher intra-abdominal pressure and a high abdominothoracic pressure gradient, which may cause gastric content to reflux up into the esophagus.^[47]^ It has also been linked to a higher incidence of hiatal hernias and subsequent functional impairment of the LES.^[47]^ Research has shown that being overweight (body mass index [BMI], 25–30 kg/m^2^) and being obese (BMI, >30 kg/m^2^) were related to GERD symptoms (OR, 1.43; 95% CI, 1.158–1.774 and OR, 1.94; 95% CI, 1.468–2.566, respectively).^[48,49]^ The researchers at^[50]^ carried out a cross-sectional study involving 206 consecutive patients who were not taking acid-suppressing medications and had undertaken a 24-hour pH measurement. The findings revealed that there was a significant (*P* < .005) relationship between a BMI > 30, high waist circumference, and the incidence of acid reflux.

Prior research has revealed personal weight increased during the pandemic.^[51]^ Bakaloudi, Barazzoni, Bischoff, Breda, Wickramasinghe and Chourdakis^[52]^ discovered that COVID-19 lockdowns caused increases in the body weight of participants ≥ 16 years old. A considerable percentage of the subjects reported that their body weight increased during lockdown, leading to significant body weight increments. Notable increases in BMI and body weight during the pandemic were also identified in a study carried out by Chang, Chen, Chen, Chen, Hsu, Chou and Chang^[53]^; and Pellegrini, Ponzo, Rosato, Scumaci, Goiter, Benso, Belcastro, Crespi, De Michieli and Ghigo.^[54]^ Moreover, the correlation between a patient elevated BMI and weight and GERD is based on the idea that similar environmental factors are to blame for the widespread occurrence of both illnesses in society. There is evidence to suggest that eating food high in fat and volume is linked to a heightened risk of GER.^[49]^ Furthermore, it has been demonstrated that high-calorie diets increase the esophagus’ exposure to acid.^[49,55]^ Thus, it stands to reason that the same eating patterns could exacerbate both conditions. Furthermore, the fact that the excess weight presses against the abdomen is a plausible explanation.^[56]^

## 4. COVID-19 stress and GER

The relationship between GER and psychological factors has been found to be significant in previous studies by modifying esophageal motility, increasing stomach acid secretion, and/or reducing pressure in the LES^[57]^ some psychological disorders (i.e., anxiety) can directly cause acid reflux.^[58]^ During psychological stress, the tight connections of the esophageal epithelium in rats were disrupted, which decreased the esophageal mucosa barrier function and made it more vulnerable to reflux.^[59]^ Furthermore, hypochondriasis, which is a result of anxiety and sadness, can lower the threshold for reflux detection and exacerbate the impression of reflux symptoms in comparison to control subjects.^[60]^ A previous study revealed that there was a significant relationship between the severity of reflux symptoms and levels of anxiety levels in patients experiencing GERD.^[61]^

Numerous studies documented the psychological toll that mandatory quarantines during earlier outbreaks took on individuals. Meanwhile, when responding to a Web-based survey, 129 patients under quarantine during the 2002 to 2004 SARS pandemic in Toronto demonstrated a significant prevalence of psychological distress, as demonstrated by Hawryluck, Gold, Robinson, Pogorski, Galea and Styra.^[62]^ According to Jeong, Yim, Song, Ki, Min, Cho and Chae^[63]^ research, 7.6% of the 1656 patients who had contact with MERS patients during the isolation period displayed symptoms of anxiety. A recent online survey carried out during the COVID-19 pandemic by Mazza, Ricci, Biondi, Colasanti, Ferracuti, Napoli and Roma^[64]^ involving 2766 Italian people highlighted a correlation between higher felt stress levels and acquaintance of COVID-19 infection. Reflux sensitivity may also result from stress.^[65]^ Thibodeau, Welch, Katz and Asmundson^[66]^ discovered that anxiety, particularly when it is caused by health-related concerns, might reduce a person pain threshold and increase awareness of reflux episodes. Furthermore, stress has been linked to increased oral intake throughout the day, which raises stomach pressure and exacerbates reflux.^[67]^

## 5. Impacts of COVID-19 on GERD esophageal mobility and function

In the previous section, it was revealed that GERD patients who did not have SARS-CoV-2 experienced heartburn and regurgitation more often even though they received the same dose of treatment during the pandemic and associated lockdowns. This was found to be related to changes in lifestyle experienced during the lockdowns.

According to earlier studies, ACE2 is the functional receptor for SARS-CoV and is essential to the virus cellular entrance.^[68]^ Additionally, a number of investigations have demonstrated that SARS-CoV-2 enters target cells by utilizing the ACE2 receptor.^[69]^ Many human organs, such as the nasal and oral mucosa, nasopharynx, esophagus, lung, small intestine, colon, kidney, spleen, liver, and brain, have large distributions of ACE2.^[70]^ Furthermore, compared to the respiratory system, ACE2 expression is said to be about 100 times higher in the GIT.^[71,72]^ Thus, the fact that the digestive system (which contains multiple organs that express ACE2) is more vulnerable to SARS-CoV-2 infection is not surprising. In turn, this could affect GI comorbidity. Moreover, although fever, cough, and dyspnea are the most common respiratory symptoms associated with COVID-19,^[73]^ a clinically significant subset of COVID-19 patients has also been reported to have stomach problems, frequently together with concurrently increased liver enzymes.^[74]^ Digestive symptoms have been recorded as the first sign of COVID-19 in certain cases.^[75]^ These results imply that the virus can affect the GIT, which could account for the variety of digestive symptoms associated with COVID-19, such as nausea, vomiting, diarrhea, and decreased appetite.

A COVID-19 gastrointestinal endoscopy was carried out by Xiao and Tang on a patient with confirmed COVID-19.^[76]^ Histological investigation revealed damage to the mucosa of the esophagus and indicated the presence of many plasma cells and lymphocytes infiltrating the stomach and duodenum lamina propria. Additionally, the research by Xiao, Tang, Zheng, Liu, Li and Shan^[76]^showed that ACE2 is present in the stratified epithelium cells of the esophagus, which may explain COVID-19-induced esophagitis and suggest a possible link between the virus and other esophageal diseases, such as GERD. Nonetheless, the processes underlying COVID-19-related esophageal injury require further investigation.

The function of the upper and lower esophageal sphincter mechanism which protect the airway from GER are mediated by multiple complex neurological reflexes. These involve the vagus and glossopharyngeal cranial nerves, in addition to the phrenic nerve, which supplies the diaphragm.^[77]^ It is interesting to note that phrenic nerve palsy has also been reported in COVID patients.^[78]^ Thus, it is reasonable to assume that these reflexes may be directly or indirectly disrupted after viral injury, which can impact the nerves involved in these neurological reflexes, which subsequently causes GER.

Additionally, there are more plausible mechanisms that need to be considered. It is widely known that symptoms similar to reflux can be caused by changes in the gut microbiome.^[79]^ According to Greenberg, Palacardo, Edelmuth, Egan, Lee, Schnoll-Sussman, Katz, Finnerty, Fahey and Zarnegar,^[80]^ 60% of patients who seek anti-reflux surgery do so because they have small intestinal bacterial overgrowth. A correlation between alterations in the GI microbiota and a number of pathologic conditions, including GERD, was discovered by Okereke, Hamilton, Reep, Krill, Booth, Ghouri, Jala, Andersen and Pyles.^[81]^

According to recent research, COVID-19 has led to the dysregulation of gut flora.^[79]^ Infection of intestinal enterocytes by SARS-CoV-2 can result in inflammation and dysbiosis of the gut.^[82]^ When there is an imbalance between beneficial and harmful bacteria in the gastrointestinal system, a disease known as dysbiosis develops.^[83]^ Additionally, a recent meta-analysis revealed that SARS-CoV-2 RNAs were found in the fecal collections of COVID-19 patients at a rate of 43.7%, with substantially higher detection rates in patients with severe infection.^[84]^ We can speculate that the gas generated by the altered COVID-19 bacteria places sufficient pressure on the stomach and small intestine to force acid into the esophagus from the stomach.^[85,86]^ Microbiome changes can cause the sugars in the small intestine to become fermented by micro-organisms. In turn, this can result in the release of intra-luminal gas and cause excessive belching.^[87]^ Reflux symptoms are believed to be caused by the belching of aerosolized gastric contents into the esophagus.^[88]^

## 6. The severity of GOR and COVID

There is only 1 existing study that has examined the prevalence of EER in patients hospitalized due to COVID-19 and the impacts that EER has on the severity of COVID-19 infection.^[89]^ Al-Momani, Mashal, Al Balawi, Almasri, AL-Shudifat, Khasawneh, Pearson and Ward^[89]^employed the reflux symptom index to evaluate the incidence of EER as well salivary pepsin levels were measured to serve as a reliable marker for EER.^[90]^ Al-Momani, Mashal, Al Balawi, Almasri, AL-Shudifat, Khasawneh, Pearson and Ward^[89]^showed that respiratory comorbidity, salivary pepsin level, and reflux symptom index score are risk factors for a patient development of a more severe COVID-19 illness. The findings of this study thus add significantly to the body of knowledge currently available regarding the effects of EER, a prevalent GI condition, on individuals with COVID-19.

The Reflex Theory and Reflux Theory are the 2 schools of thought that now attempt to explain why COVID-19 infection severity increases in patients with end-of-range symptoms.^[91]^ Under the Reflex Theory, it is assumed that gastric reflux in the esophagus stimulates the vagus nerve, resulting in bronchospasms. The bronchial hyper-responsiveness subsequently rises as a result, negatively affecting the airways.^[92]^ Conversely, the Reflux Theory postulates that aspiration of gastric refluxate results in airway inflammation and injury, which exacerbates the deleterious effects of COVID-19.^[93]^

## 7. COVID-19 and LPR and potential aspiration

Although coughing and shortness of breath are the primary respiratory symptoms of COVID-19,^[94]^ there is increasing evidence that the virus is a multi-system illness that also manifests with non-respiratory symptoms. Additionally, the virus has been found to impact the GIT as previously mentioned, causing symptoms such as nausea, diarrhea, and loss of appetite.^[95,96]^ The US Centers for Disease Control and Prevention (CDC) produced a recent report indicating that people suffering from severe COVID-19 have the infectious virus in their feces and not simply viral RNA.^[97]^ Likewise, a similar study reported the presence of the “live” virus in human feces.^[98]^ Other reports have suggested that viral RNA is present in the stools of 50% of COVID-19 patients, even when the virus is not concurrently present in their respiratory tract.^[99]^ This research, combined with other studies,^[100,101]^ strongly indicates that the GI system serves as a central hub for SARS-CoV-2 infection. There is also a possibility of fecal-oral transmission.^[96]^

Thus, aspiration via EER is an additional mechanism by which COVID-19 may enter individual lungs from the GIT (Gut-lung axis). Previous research using culture-independent techniques has demonstrated that there is microbiological continuity in healthy adults’ aerodigestive tracts, suggesting that the microaspiration of gastric content could be prevalent even in healthy people.^[102]^ Additionally, earlier studies have highlighted a relationship between EER and a number of respiratory tract problems, including wheezing, post-nasal drip, coughing, sore throats, tight chests, and continuous throat clearing.^[103]^ furthermore, EER has been linked to otitis media and tooth decay, with cases of refluxate entering the respiratory passages reported.^[104,105]^

Numerous studies have also shown that the stomach reservoir increases the possibility of developing nosocomial pneumonia during hospitalization at intensive care units.^[106]^ In our earlier work, we focused on the stomach function as a reservoir for bacterial infections.^[107,108]^ Moreover, a bacterial relationship has been found between the respiratory tract and the gut in other studies conducted with elderly participants. To be more precise, the same bacteria were found in large concentrations in the gut before they were found in the respiratory tract.^[109]^ This indicated a clear connection between gastrointestinal and lower respiratory tract bacterial colonization,^[110]^ which may also be true for viral infection and transmission. It has been found that 6 out of 13 severely ill patients in an early pandemic trial had gastric fluid that tested positive for SARS-CoV-2 by PCR,^[111]^ and SARS-CoV-2 can survive in simulated intestinal and stomach fluids with a pH range of 4 to 7.^[112]^ This pH range (4–7) generally happens after a person has consumed a regular meal.^[113]^

In our previous study, a relationship was identified between gastric juice microflora and sputum samples from individuals with cystic fibrosis. This suggests that there may be a significant gastric infection etiology in cystic fibrosis, as well as a potential microbe reservoir.^[108,114]^ Moreover, Palm, Sawicki and Rosen^[115]^ conducted research into cystic fibrosis cases in children and found evidence to support the existence of a relationship between lower-airway *pseudomonas aeruginosa* infection and GER, which further supports the Theory of Microbial Transfer between the respiratory and gastrointestinal systems. A study conducted recently also showed that there may be a microbial translocation between the GIT and the lungs, which is independent of the ecology of the oropharyngeal tract.^[116]^ In this particular research, 8 of the most frequently identified bacteria in gastric fluid samples were also found to be present in the lungs in children suffering from chronic cough.^[116]^ Further evidence provided by Blake and Teague^[117]^ who found that intestinal and respiratory infections were more common in children with GERD who were treated with proton-pump inhibitors (PPI) or H2 blockers.^[117]^ This may be explained by the survival of bacterial in the stomach.

Similarly, previous consumption of PPIs has been associated with an increased risk of COVID-19-related death, as demonstrated by Almario, Chey and Spiegel^[118]^. A meta-analysis of 8 studies^[119]^ showing that prior PPI use raises the likelihood of COVID-19 development to a severe manifestation corroborated this association. In addition, PPI users had a greater likelihood of testing positive for COVID-19 than non-users Almario, Chey and Spiegel.^[118]^ Their theory was that by weakening the stomach barrier, taking PPIs can increase the risk of contracting COVID-19.

PPIs primarily work by permanently inhibiting the proton-pump, which suppresses the production of stomach acid.^[120]^ Hypochlorhydria can reduce the protective function of stomach acid, which can help SARS-CoV-2 survive in the stomach and make it easier for enterocytes to invade. This can also apply to direct viral-lung interaction by gastric reflux and aspiration. As a result, taking PPIs may expose the lungs to live COVID-19 viruses and leave the stomach open to coronavirus infection. According to Xiao, Chakraborti, Dimitrov, Gramatikoff and Dimitrov,^[121]^ under neutral pH environments, the S protein of SARS-CoV facilitated fusion with host cells. Meanwhile, Darnell, Subbarao, Feinstone and Taylor^[122]^ confirmed that a highly alkaline pH of 12 to 14 and a highly acidic pH of 1 to 3 can cause SARS-CoV to become inactivated, although the virus can remain stable within the neutral pH range. In a similar fashion, SARS-CoV-2 can survive for 6 days, although it will lose between 2.9 and 5.33 logs of infectivity if sustained at a pH level of 5 to 9.^[122]^ Zhou, Niu, Jiang, Zhang, Zheng, Wang, Zhu, Gao, Huang and Wang^[123]^ found that, at extreme pH levels (pH 2–3 and pH 11–12), SARS-CoV-2 lost infectivity within 24 hours. They also found that SARS-CoV-2 was fully inactivated and incapable of infecting cells when kept at pH levels of 1.0 and 2.0 (akin to the normal acidity of an empty stomach) by pseudotyping viruses with the SARS-CoV-2 S protein. The pH of the small and large intestines is between 7.5 and 8.0, but the pH of stomach secretions ranges from 1.0 to 3.5. The majority of SARS-CoV-2 virus particles may be rendered inactive by stomach acid when consumed. However, a person stomach acidity will decrease if they take an acid suppressant, like PPI, for an extended length of time. Thus, there may be a greater possibility for SARS-CoV-2 to get from the stomach into the gut, where it could cause a viral infection and raise the possibility of GI symptoms.^[124]^ This is on top of the virus heightened capacity to survive in low-acid environments, which increases the risk of respiratory system reflux. It is obvious that additional research is required to ascertain how PPIs affect SARS-CoV-2 survival in the gastrointestinal environment. This is especially crucial because low stomach acid, or hypochlorhydria, is very common in the elderly,^[125]^ a population that is much more likely to contract the virus.

It is widely known that COVID-19 infection has a more profound impact on the older population. Many elderly persons have a higher gastric pH than normal, which might be caused by atrophic gastritis or drugs that reduce acidity.^[126]^ Children are often free of severe COVID-19 infection because of their normal gastric acid levels.^[127,128]^ These variables may influence an individual vulnerability to COVID-19 infections, at least partially.

## 8. Conclusion

As well as causing severe respiratory pathology, the novel SARS-CoV-2 virus can cause GI manifestations. The immunopathological effects of COVID-19 and SARS-CoV-2 replication are likely reflected in the GI dispersion. Unquestionably, there is direct viral infectivity and toxicity due to the co-expression of ACE2 and transmembrane serine protease 2 in the GI and systems, the local identification and replication of SARS-CoV-2, and the disorders of the gut and liver when no respiratory symptoms are present. The GI system might serve as a hub for SARS-CoV-2 invasion and spread. As far as the researchers are aware, this is the first review that discusses how a lockdown measurement and COVID-19 infection affect GERD patients’ stomach reflux. The impact that lockdowns had on GERD patients’ stress levels and eating patterns. Both variables have been found to increase GERD symptoms.

At present, it is unknown whether COVID-19 infection causes esophageal neurological or physiological injury that enhances the prevalence of GERD symptoms. However, the gastrointestinal symptoms associated with COVID-19 have been well documented. There are still questions regarding the precise pathophysiological relationship between COVID and GERD, and it possible that a number of different processes could be at play when reflux symptoms arise during COVID-19. In order to fully understand the mechanisms underlying COVID-19 effects on the neurological system, cranial nerves, and the neural responses that often prevent GER, more research is required.

Determining the processes underlying the relationship between gastric reflux and COVID-19 lung disease is a challenging task due to the intricate interactions between the GIT and the respiratory system. The interaction of structural defects and/or physiological dysfunction in the lungs, esophagus, stomach, and brain likely determines whether reflux exacerbates COVID-19 or whether acute COVID-19 infection amplifies reflux. Nonetheless, there are still a few areas that require further investigation, including the importance of these processes for individual patients, the misalignment that they create between the esophagus and airways, and the coexistence of reflux and pulmonary disease.

## Author contributions

**Conceptualization:** Hafez Al-Momani.

**Resources:** Hafez Al-Momani.

**Writing – original draft:** Hafez Al-Momani, Iman Aolymat, Sameer Al Haj Mahmoud.
